# ASIC1 and ASIC3 mediate cellular senescence of human nucleus pulposus mesenchymal stem cells during intervertebral disc degeneration

**DOI:** 10.18632/aging.202850

**Published:** 2021-04-06

**Authors:** Jingyu Ding, Renjie Zhang, Huimin Li, Qiang Ji, Xiaomin Cheng, Rick Francis Thorne, Hubert Hondermarck, Xiaoying Liu, Cailiang Shen

**Affiliations:** 1 Department of Orthopedics and Spine Surgery, The First Affiliated Hospital, Anhui Medical University, Hefei 230032, Anhui, China; 2 School of Life Sciences, Anhui Medical University, Hefei 230032, Anhui, China; 3 Translational Research Institute of Henan Provincial People’s Hospital and People’s Hospital of Zhengzhou University, Molecular Pathology Centre, Academy of Medical Sciences, Zhengzhou University, Zhengzhou 450053, Henan, China; 4 School of Biomedical Sciences and Pharmacy, University of Newcastle, Callaghan, NSW 2308, Australia

**Keywords:** intervertebral disc degeneration (IVDD), nucleus pulposus mesenchymal stem cells (NP-MSCs), acid sensing ion channels (ASICs), cellular senescence

## Abstract

Stem cell approaches have become an attractive therapeutic option for intervertebral disc degeneration (IVDD). Nucleus pulposus mesenchymal stem cells (NP-MSCs) participate in the regeneration and homeostasis of the intervertebral disc (IVD), but the molecular mechanisms governing these processes remain to be elucidated. Acid-sensing ion channels (ASICs) which act as key receptors for extracellular protons in central and peripheral neurons, have been implicated in IVDD where degeneration is associated with reduced microenvironmental pH. Here we show that ASIC1 and ASIC3, but not ASIC2 and ASIC4 are upregulated in human IVDs according to the degree of clinical degeneration. Subjecting IVD-derived NP-MSCs to pH 6.6 culture conditions to mimic pathological IVD changes resulted in decreased cell proliferation that was associated with cell cycle arrest and induction of senescence. Key molecular changes observed were increased expression of p53, p21, p27, p16 and Rb1. Instructively, premature senescence in NP-MSCs could be largely alleviated using ASIC inhibitors, suggesting both ASIC1 and ASIC3 act decisively upstream to activate senescence programming pathways including p53-p21/p27 and p16-Rb1 signaling. These results highlight the potential of ASIC inhibitors as a therapeutic approach for IVDD and broadly define an *in vitro* system that can be used to evaluate other IVDD therapies.

## INTRODUCTION

Low back pain (LBP) is a leading cause of disability which affects more than 70% of the worldwide population [1]. Analysis of records from 1997 to 2017 shows LBP has been a common symptom that presents as a debilitating health problem that carries very high socioeconomic burden in all countries [2, 3]. Although many potential factors have been found to be related to LBP, intervertebral disc degeneration (IVDD) is one of the most important [4]. The intervertebral disc (IVD) is the key component of human spine which allows movement and links the superior and inferior vertebral bodies via the cartilaginous endplates (CEP) and is composed of three distinct components: the central highly hydrated gelatinous nucleus pulposus (NP), highly organized peripheral annulus fibrous (AF) and the CEP.

IVDD is known to involve both age-related changes and tissue damage caused by diverse stresses [5], and is characterized by chronic decreases in the number and function of NP cells along with associated loss of extracellular matrix (ECM). Understanding of these fundamental changes has focused attention towards biological treatments that facilitate the recovery of ECM function including gene therapy and other means to enable disc regeneration such as stem cell therapies [6]. Indeed, prior research has identified that nucleus pulposus mesenchymal stem cells (NP-MSCs) exist within the IVD [7] and that these cells participate in the regeneration and homeostasis of the IVD [8]. These endogenic stem cells self-renew and differentiate into NP cells that are responsible for self-regeneration and when activated can promote regeneration of the ECM [9].

The IVD represents the largest non-vascular tissue in the body [10]. Its main energy supply comes from the capillaries of the vertebral bodies, which nourish NP cells by diffusion through the cartilage endplate [5] but this process is inhibited when the disk and cartilage endplates undergo degeneration [11]. This leads to the changes in the IVD microenvironment through the accumulation of metabolites leading to hypoxia and acidosis (low pH) [12]. Regarding the latter, the pH of healthy discs is 7.1 [13] but it drops to 6.5 or even lower in severely degenerated discs [14, 15]. Importantly, an acidic microenvironment plays a key role in IVD degeneration since it causes loss of chondrocytes and consequent reductions in ECM [16, 17].

Acid-sensing ion channels (ASICs), a membrane H^+^-gated subgroup of the degenerin/epithelial Na^+^ channel (DEG/ENaC) family, have emerged as key receptors for extracellular protons in central and peripheral neurons. Four genes (Accn1, Accn2, Accn3 and Accn4) encode at least seven ASIC isoforms including ASIC1a, ASIC1b, ASIC1b_2_, ASIC2a, ASIC2b, ASIC3, ASIC4 [18–20], and each ASIC is comprised of three subunits which can form functional homo- or hetero- trimeric channels [21]. ASICs are widely distributed in various tissues of mammals [22–24] and exert diverse roles in acid sensation, taste, learning and memory, proprioception, Na/H_2_O transport and mechanosensation in many tissues. ASICs are expressed in nervous and joint tissues and have been implicated in neurodegenerative diseases including spinal cord injury, multiple sclerosis, Parkinson’s disease and Huntington’s disease [25–30]. Notably, ASICs are activated by acidosis, lactate and arachidonic acid and highly acidic conditions increase ASIC expression to promote survival of NP cells [31]. Instructively, blockade of ASICs by Amiloride, Psalmotoxin 1 (PcTx1), APETx2 or specific small interfering RNA (siRNA) can restrain acid-induced apoptosis in endplate chondrocytes of IVDs [32–35]. These results serve to illustrate the connection between acidic pH and ASIC function during IVD degeneration. However, the functional status of ASICs in NP-MSCs, a critical cell population required for IVD maintenance and regeneration, remains unclear.

In this report we studied the expression of ASICs in the IVD with different degrees of degeneration together with the interrelationship between ASIC function and acidosis. We found ASIC1 and ASIC3 were selectively increased in freshly isolated IVD tissues according to their degree of degeneration and that the physiological upregulation of ASIC1/ASIC3 could be mimicked *in vitro* by subjecting NP-MSCs to acidic culture conditions to simulate acidosis. Moreover, such treatment led to cell cycle and entry into cellular senescence [36–39] and we identified a number of potential senescence programming pathways involved. Consistently, we demonstrate that ASIC inhibitors prevent senescence induction in NP-MSCs, a finding which supports their potential as therapeutics for IVDD.

## RESULTS

### Characterization of nucleus pulposus stem cells

**Figure 1:**
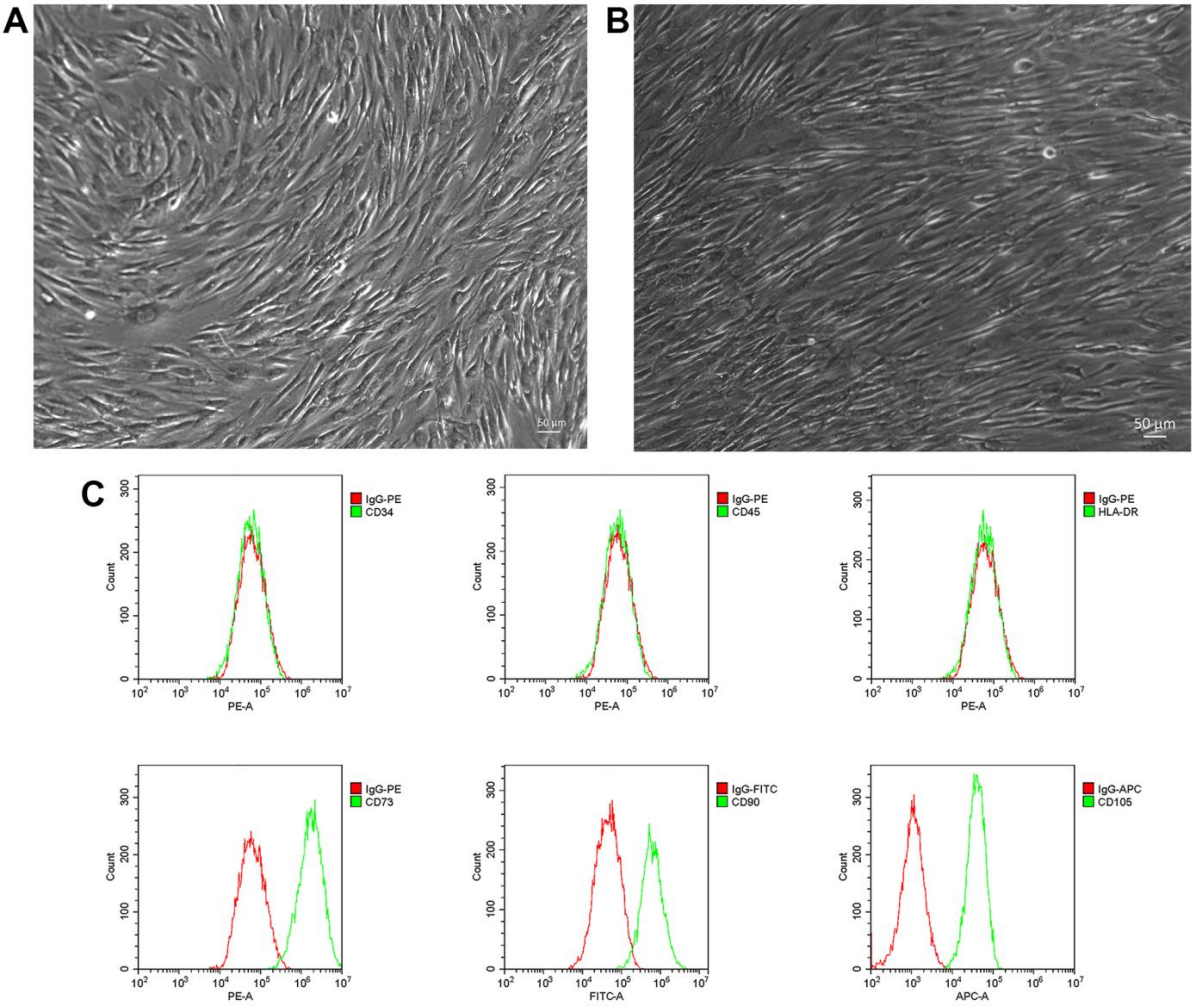
**Isolation and phenotyping of *in vitro* cultured NP-MSCs.** Representative phase-contrast images of passage 1 (**A**) and passage 2 (3 weeks) (bar = 50 μm) (**B**) NP-MSCs showing adherent cells with a long-spindle shape morphology. (**C**) Analyses of cell surface markers of NP-MSCs by flow cytometry, showing positive CD105, CD90, CD73 and negative CD34, CD45 and HLA-DR. All the samples were from case 5.

Primary NP-MSCs isolated from human degenerated IVDs were cultured with low-glucose DMEM medium and displayed the morphological appearance of long-spindle shape cells that were strongly adherent to the plastic substrate (Figure 1A). The cells reached ~80% confluence approximately three weeks later and maintained a consistent appearance (Figure 1B). The identity of the cultured cells from IVDDs was confirmed by flow cytometric analyses demonstrating high expression of CD105, CD90 and CD73, with very low or negative expression of CD34, CD45 and HLA-DR (Figure 1C), consistent with the known phenotypes of NP-MSCs [40].

### ASIC1 and ASIC3 are upregulated in human NP-MSCs with different degrees of degeneration

**Figure 2:**
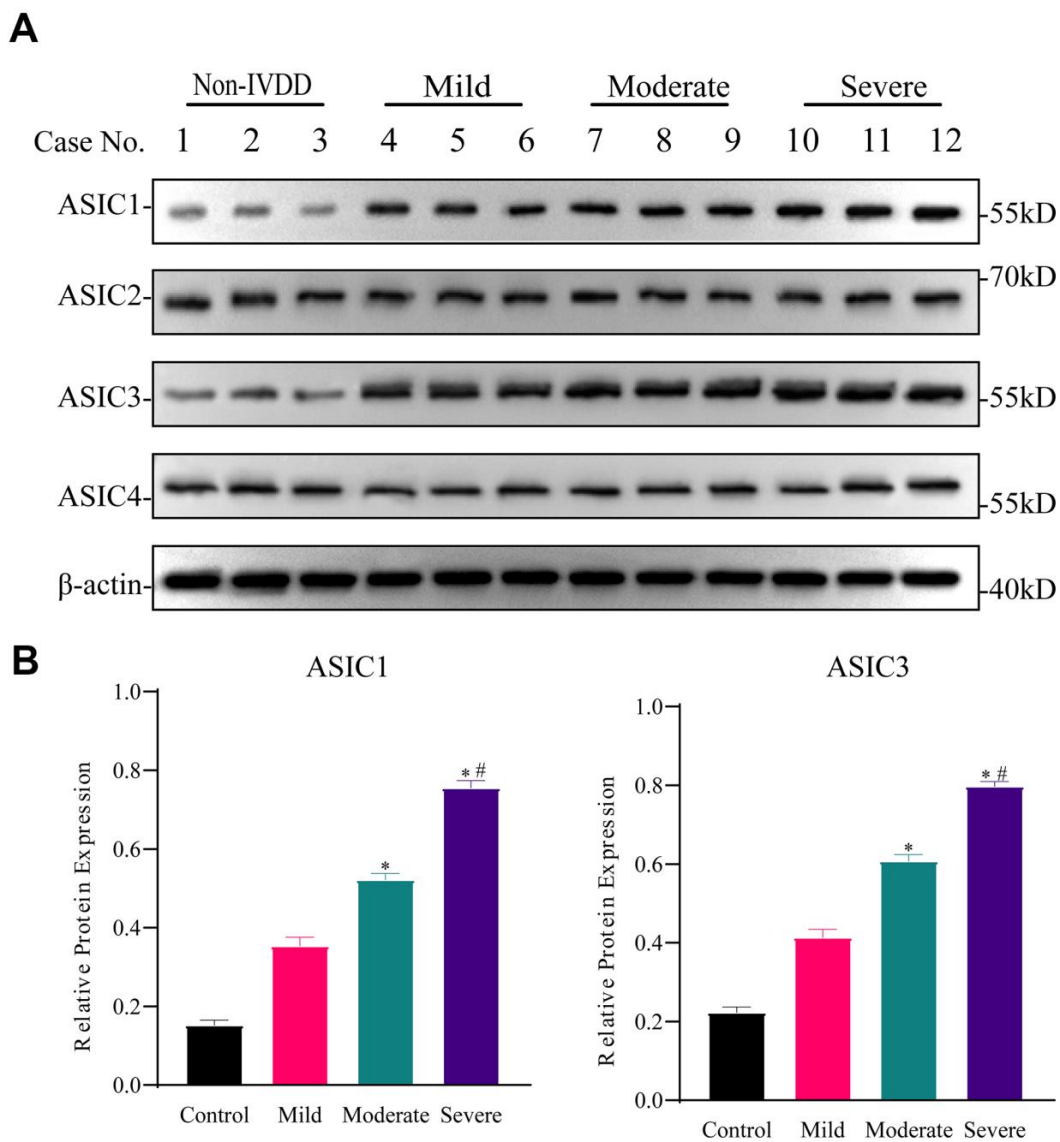
**Differential expression of ASICs in freshly isolated human IVD tissues with different degrees of intervertebral disc degeneration (IVDD).** (**A**) Representative Western blotting analyses of ASICs 1-4 in IVD tissues from non-IVDD and IVDD groups from mild to severe cases. (**B**) Analyses of the relative expression of ASIC1 and ASIC3 determined for all patient IVD tissues (n=3 for each group) using densitometric analysis of Western blotting as per (**A**). Data are mean ± SD. * indicates P≤0.05 compared to non-IVDD group; # indicates P≤0.05 compared to mild group.

We investigated the expression of the ASIC isoforms, ASIC1, ASIC2, ASIC3 and ASIC4 in primary IVD isolates with differing degrees of degeneration. Western blotting analyses showed that the expression levels of ASIC1 and ASIC3 were progressively upregulated in NP-MSCs derived from mild (Pfirrmann grade II), moderate (Pfirrmann grade III) through to severely degenerated IVDs (Pfirrmann grade IV) compared with non-IVDD derived aspirate biopsy samples (Figure 2A, 2B and Supplementary Figure 1). Thus, there are specific effects on ASIC isoforms, a finding which is consistent with a prior report showing that ASIC1 and ASIC3 are most sensitive to H^+^ [21].

### Acidosis-induced upregulation of ASIC1 and ASIC3 can be reversed by ASIC inhibitors

**Figure 3:**
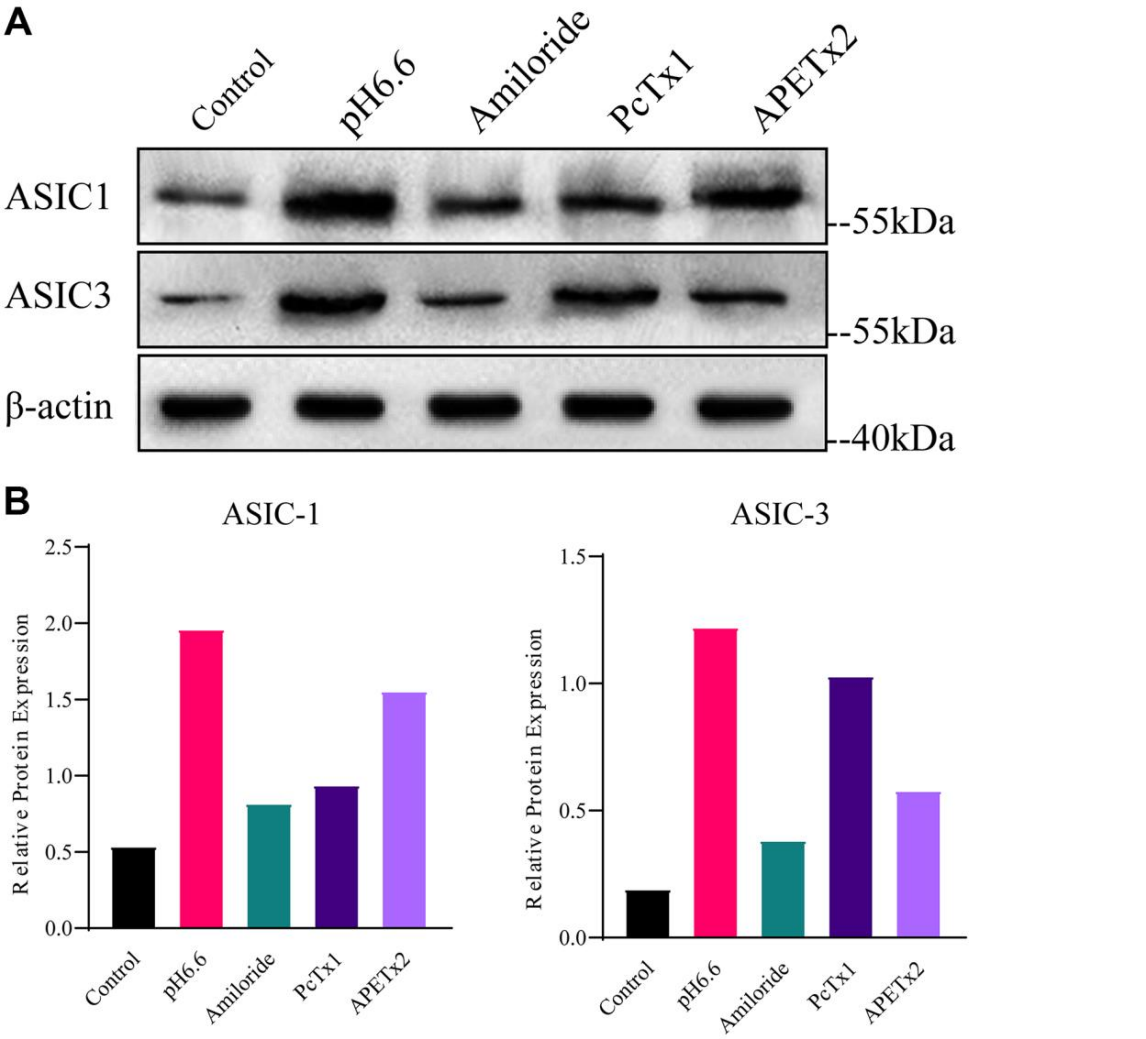
**Analyses of ASIC1 and ASIC3 expression in primary cultured human NP-MSCs.** (**A**) Western blotting analyses of ASIC1 and ASIC3 expression when exposed to extracellular acid (pH6.6) with or without treatment of inhibitor Amiloride, PcTx1 or APETx2. (**B**) The relative expression of ASIC1 and ASIC3 using densitometric analysis of specific bands revealed by Western blotting as per (**A**). The samples were from case 4. Data are mean ± SD. With * indicating P≤0.05.

To investigate the potential of acidic microenvironmental conditions to upregulate ASIC1 and ASIC3 in NP-MSCs, we cultured isolated cells under pH 6.6 conditions that mimic the degenerated intervertebral disc [17]. In concert we also evaluated the effects of the non-specific ASIC inhibitor Amiloride in addition to PcTx1 and APETx2 which are specific inhibitors of ASIC1 and ASIC3, respectively. Instructively, NP-MSCs grown under acidic conditions displayed increased expression of both ASIC1 and ASIC3 whereas treatment with Amiloride and PcTx1, and to a lesser extent APETx2, prevented acid-induced increases in their expression (Figure 3A, 3B and Supplementary Figures 2–4). These results support the notion that acidosis triggers upregulation of ASIC1 and ASIC3 in NP-MSCs and that this can be targeted using ASIC inhibitors.

### ASIC inhibitors rescue the acidosis-induced decline in NP-MSC proliferation

**Figure 4:**
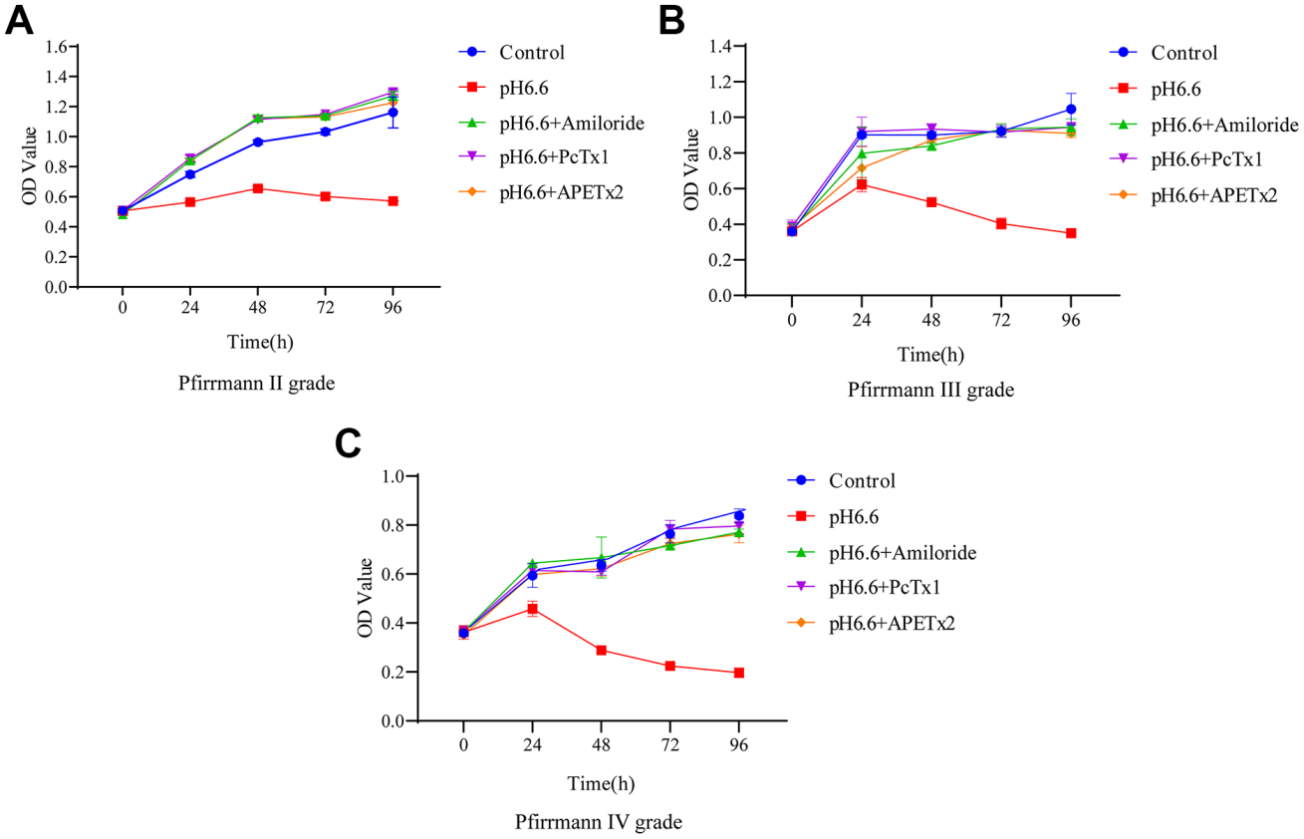
**The effect of acidic culture conditions on the proliferation of human NP-MSCs.** The proliferation ability of human NP-MSCs derived from patients with Pfirrmann grade II (**A**), III (**B**) and IV (**C**) degeneration was determined using CCK-8 assays under control conditions or after exposure to extracellular acid (pH6.6) alone and in combination with addition of the inhibitors Amiloride, PcTx1 or APETx2. n=3 for each group; data are mean ± SD.

Our previous work indicated that the proliferation rate of NP-MSCs decreases under acidic conditions [17]. Given the degeneration associated changes in ASIC1 and ASIC3 expression by NP-MSCs, in particular the increases triggered by acidic conditions, we considered that ASIC1 and ASIC3 may play a vital pH sensing role that affects NP-MSC proliferation. To test this notion, we took advantage of the ability of ASIC inhibitors to prevent upregulation of ASIC1 and ASIC3 triggered by acidosis. Measuring NP-MSC proliferation using CCK-8, an assay which uses cellular dehydrogenase activity as a proxy measure, showed that Pfirrmann grade II and grade IV isolates failed to grow at pH 6.6 in contrast to normal culture conditions (Figure 4A–4C). Importantly, treating the cells with ASIC inhibitors (Amiloride, PCTx1 and APETx2) served to restore the proliferation capacity of NP-MSCs to normal levels. Together these data suggest that inhibition of proliferation of NP-MSCs in the acidic microenvironment involves the functions of ASIC1 and ASIC3.

### Acidosis induces cell cycle arrest and senescence in NP-MSCs which can be prevented by ASIC inhibitors

**Figure 5:**
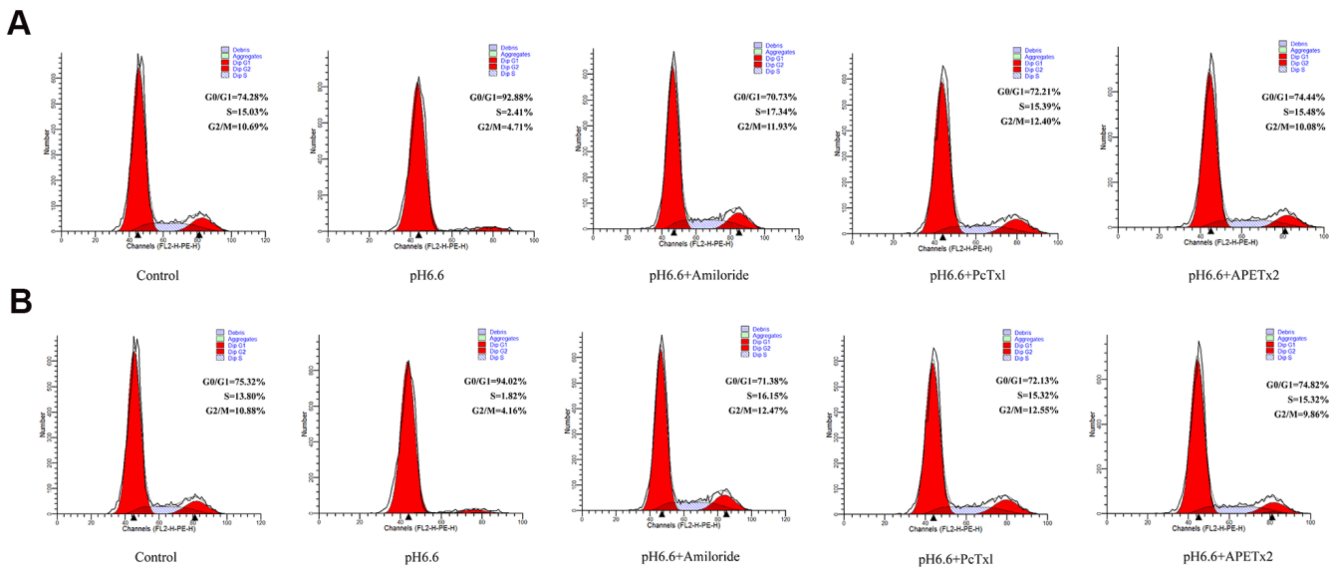
**Acidic culture conditions inhibit cell cycle progression in human NP-MSCs which can be reversed using ASICs inhibitors.** Flow cytometric DNA content profiles comparing cultured NP-MSCs derived from Pfirrmann grade II (**A**) and IV (**B**) cases cultured under control or acidic pH conditions in combination with or without treatment using Amiloride, PcTx1, APETx2, respectively. G0/G1, S and G2/M cell cycle phases were estimated as % totals using the ModFit software. The samples in (**A**) were from case 6 and that in (**B**) from case 11.

To better understand why the population of NP-MSCs declined following culture in acid-conditioned media we sought to understand the processes involved. We considered the possibility that the cells underwent cell cycle arrest and subsequently became senescent. The impact of pH 6.6 culture on cell cycle phases was first evaluated by flow cytometry. Comparing NP-MSCs derived from Pfirrmann II grade IVDs under normal versus acidic conditions showed the numbers of proliferative cells (S+G2/M phases) substantially declined from 25.72 to 7.12% (Figure 5A). As anticipated from the proliferative assays, treatment of the NP-MSCs with the ASIC inhibitors, Amiloride, PcTx1 and APETx2 reversed the cell cycle phase changes with the numbers of proliferating cells determined as 29.27%, 27.79% and 25.56%, respectively. A comparable experiment conducted in NP-MSCs derived from a Pfirrmann IV grade case demonstrated similar findings albeit the basal proliferation rates of the cells was comparably less (Figure 5B). These data demonstrate that there is a significant population of cultured NP-MSCs that undergo cell cycle arrest.

**Figure 6:**
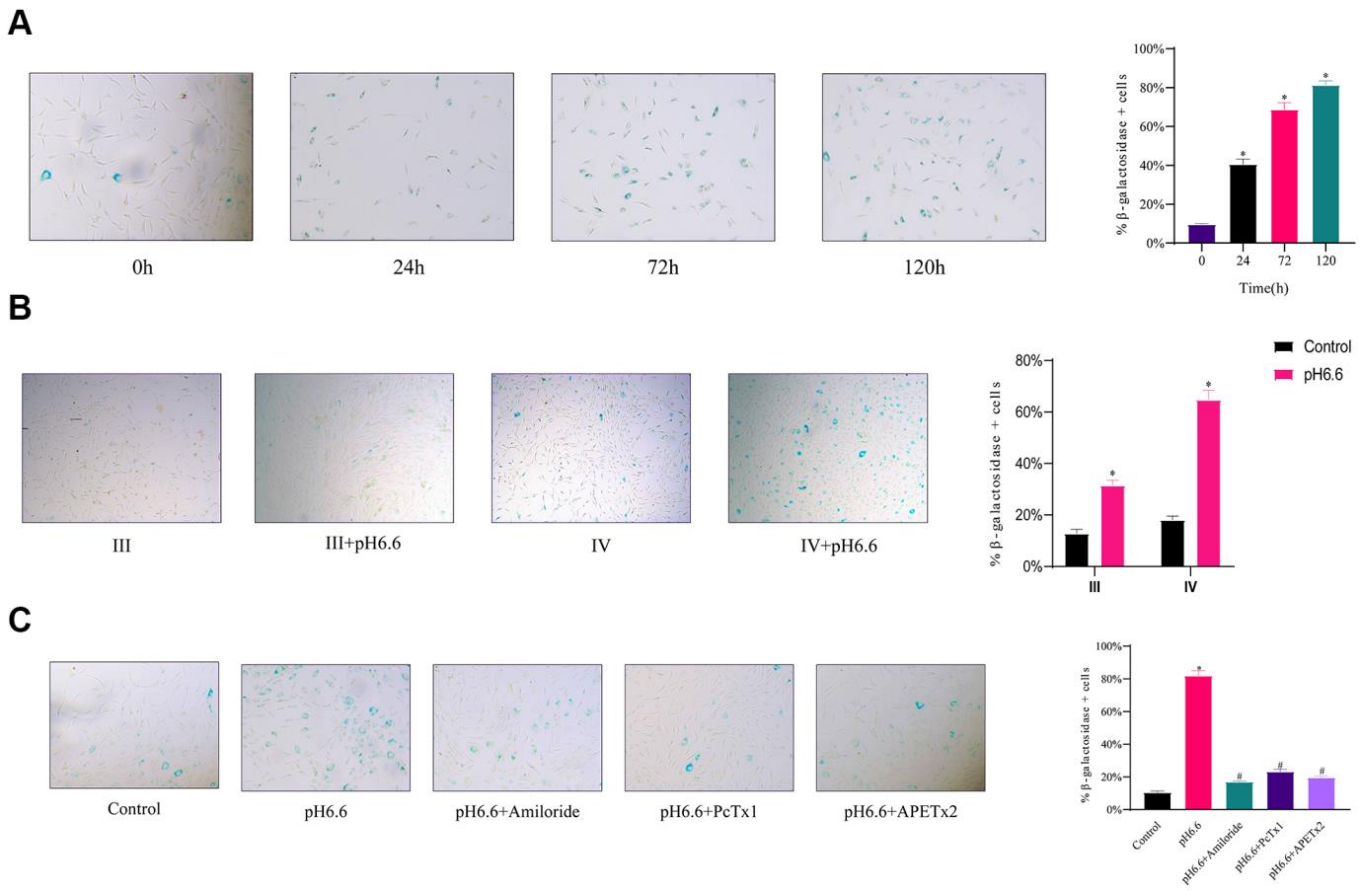
**The induction of cellular senescence in human NP-MSCs exposed to acidic conditions can be reversed by ASICs inhibition.** (**A**) Representative images (left) and the calculated percentage (right) of cultured Pfirrmann grade IV NP-MSCs undergoing senescence determined using SA-β-gal staining after exposure to acidic pH 6.6 conditions for 0, 24, 72 and 120 hours. n=3; data are mean ± SD, * P≤0.05 compared to 0 h group. (**B**) Comparative rates of senescence determined as per (**A**) in NP-MSCs derived from patients with Pfirrmann III and IV grades IVDD cultured under control or acidic pH conditions (pH 6.6) for 72 h. n=3; data are mean ± SD, * P≤0.05 compared to corresponding control group. (**C**) Effects of ASIC inhibitors on senescence induction in Pfirrmann grade II NP-MSCs cultured in control versus pH 6.6 for 120 h with or without treatment using Amiloride, PcTx1, APETx2 as indicated. The samples in (**A**) were from case 10, that in (**B**) of Pfirrmann III and IV group from case 7 and 11, respectively, and that in (**C**) from case 6. n=3; data are mean ±SD, * indicates P≤0.05 compared to control group; # indicates P≤0.05 compared to pH 6.6 group.

To ascertain if the arrested cells were entering senescence, we conducted senescence-associated β-galactosidase (SA-β-gal) staining of NP-MSCs cultures. Notably, acidic culture resulted in a time dependent increase in the percentage of senescence cells, reaching a striking ~80% of cells after 5 days (Figure 6A). Furthermore, comparing cultures of Pfirrmann stage III versus stage IV cultures showed the levels of senescent cells were double in the stage IV cells after 3 days of acidic culture (Figure 6B). Moreover, treating cells with ASIC inhibitors (Amiloride, PcTx1 or APETx2) resulted in significant reductions in the proportion of senescent cells induced by acidosis (Figure 6C).

**Figure 7:**
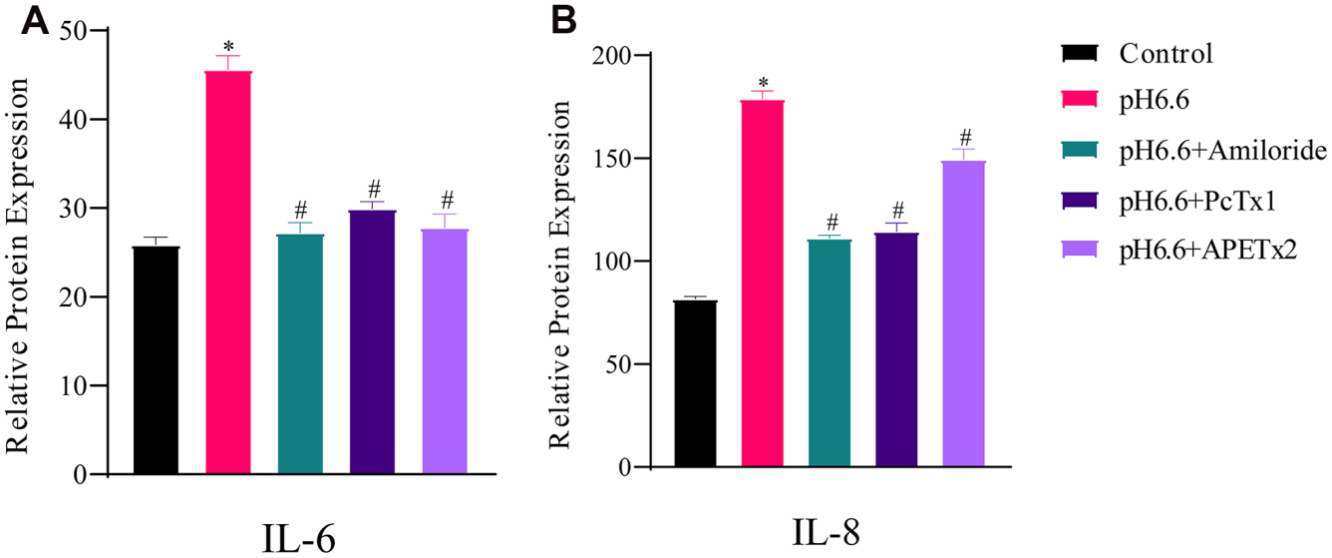
**The production of secreted SASP cytokines by human NP-MSCs is increased by acidic culture and partially reversed by ASICs inhibition.** ELISA assays measuring secreted IL-6 (**A**) and IL-8 (**B**) by human NP-MSCs after culture in control or acidic medium (pH 6.6) with or without treatment using the inhibitors Amiloride, PcTx1 or APETx2 as indicated. n=3; data are mean ±SD, * indicating P≤0.01 compared to control group at the same time points; # indicating P≤0.01 compared to pH 6.6 group at the same time points. The samples were from case 8.

As confirmation that NP-MSCs exposed to acidic conditions were senescent, along with measuring the secreted levels of IL-6 and IL-8, inflammatory cytokines implicated in the senescence-associated secretory phenotype (SASP) [41, 42]. IL-6 and IL-8 are considered the most robust SASP markers and are involved in senescence-induced growth arrest through both autocrine and paracrine mechanisms [42, 43]. Examining the relative expression levels of IL-6 and IL-8 secreted in the NP-MSC culture medium by quantitative ELISA revealed significantly increased levels of secreted IL-6 and IL-8 by NP-MSCs under pH 6.6 culture conditions (Figure 7A, 7B). As anticipated, ASIC inhibitor treatment reversed the increases in cytokine expression.

Together these data indicate that acidosis inhibits NP-MSC proliferation through inducing cell cycle arrest and senescence, and that inhibiting ASIC1 and ASIC3 can prevent cells undergoing senescence.

### Molecular profiling of protein expression in senescent NP-MSCs and the impact of ASICs

**Figure 8:**
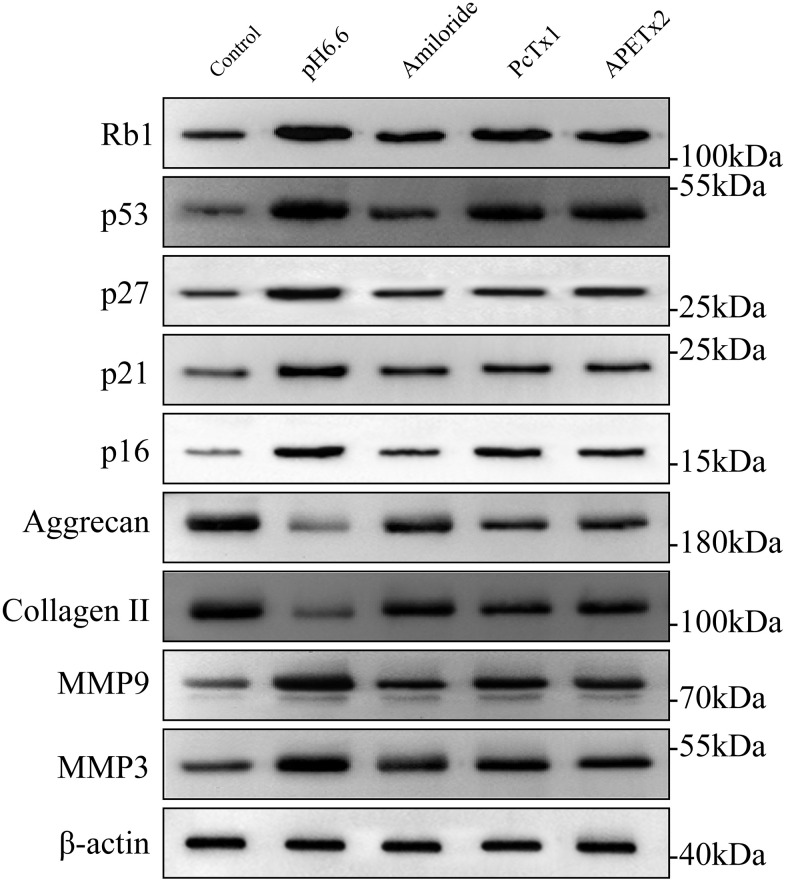
**The effect of acidic culture conditions on senescence-related protein expression in primary cultured human NP-MSCs.** Western blotting analyses were performed against Rb1, p53, p27, p21, p16, Aggrecan and Collagen II, MMP9 and MMP3 expression in Pfirrmann grade III NP-MSCs exposed to pH 6.6 culture conditions with or without treatment using the inhibitors Amiloride, PcTx1 or APETx2, respectively. The samples were from case 5, 9 and 12, respectively.

The entry of cells into senescence involves the upregulation and activation of transcription factors responsible for driving networks of genes involved in cell cycle arrest and senescence programming. These include p53 and its downstream targets p21 [44], p27 along with p16 and phosphorylated Rb1 [45, 46]. Other protein markers of interest to IVDD also include ECM components such as aggrecan and collagen II along with matrix metalloproteinases such as matrix metalloproteinase 3 and 9 (MMP3 and MMP9) which degrade multiple ECM components [47] To uncover which of these molecular mechanisms were relevant to the effects of acidosis on NP-MSCs we measured the expression of these proteins using Western blotting. Exposure of isolated NP-MSCs to acidic growth conditions resulted in relatively increased levels of p53, p21, p27, Rb1 and p16 proteins (Figure 8 and Supplementary Figures 5–7). Similarly, MMP3 and MMP9 levels also increased whereas aggrecan and collagen II levels decreased. The levels of an actin loading control protein were similar for each sample. Comparing cells treated with ASIC inhibitors showed their effectiveness at reversing the acid-induced changes to varying extents with Amiloride being the most efficient compared to the specific blockers PcTx1 and APETx2.

## DISCUSSION

In recent years, stem cell therapeutic approaches have become an attractive option for addressing the clinical problem of disc degeneration. The first reported isolation of MSC-like cells from degenerated IVDs by Risbud et al. [48] indicated the potential of employing stem cells for such therapies. Adding to the characterization of these cells, our previous research has established that IVD-isolated NP-MSCs have osteogenic, adipogenic and chondrogenic differentiation potential [17]. Other researchers including Fehlings, Huang and Zhang’s groups have all found that the NP-MSCs in intervertebral disc represent a readily accessible source for repair and regeneration [49–51]. Similarly, the means to activate or preserve endogenous NP-MSCs in the IVD represents a comparable strategy to stem cell replacement. Therefore, knowledge of the molecular mechanisms and downstream pathways affecting NP-MSCs during IVDD is crucially important in developing approaches that help maintain IVD homeostasis.

The pH in most human IVD decreases gradually with the age increase and degenerative processes [52]. The acidic environment causes physiological changes in intervertebral disc cells [5]. ASICs affect the biological activity of cells by regulating the flow of ions inside and outside of the cells [21] with a number of reports suggesting these play important roles in IVDD. Indeed, *in vitro* studies have shown that ASIC channels mediate an increase in intracellular Ca^2+^ of chondrocytes upon acidosis, and the abnormal increases in Ca^2+^ may result in cell death by apoptotic or pyroptotic mechanisms [32, 53]. *In vitro* animal experiments have observed that ASIC1 mediates cartilage endplate apoptosis and matrix metabolism under acidic conditions [54, 55]. Gilbert et al [12] also found that ASIC3 may be a potential therapeutic target for treatment of IVD degeneration. To better resolve which of these ASICs were implicated during IVDD we surveyed their expression.

Herein, we found there was progressively higher expression of ASIC1 and ASIC3, but not ASIC2 and ASIC4, in IVD tissues during IVDD ranging from mild to severe degeneration. According to our previous research, pH 6.6 culture conditions mimic the acidic microenvironment in the IVD without induction of cell death [56]. Applying this approach, we confirmed there was differential activation of ASIC1 and ASIC3 in IVD-isolated NP-MSCs. Collectively this supports the notion that acidosis was responsible for the changes in ASIC expression *in vivo*. This is the first report to definitively show that ASIC1 and ASIC3 are the key mediators of cellular senescence in human NP-MSCs during IVDD.

We had previously shown that exposing NP-MSCs to acidic conditions resulted in decreased cell proliferation [17] and we sought to expand on the underlying mechanisms, particularly the role played by ASICs. We showed cultured NP-MSCs exposed to an acidic microenvironment displayed a shift in the proportion of cells in the G0/G1 phase, indicative of cell cycle arrest which in turn is a prerequisite for entry of cells into senescence. Indeed, we showed that acidic culture conditions caused a remarkably high proportion of NP-MSCs to become senescent as scored by their expression of SA-β-gal activity and elevated expression of SASP cytokines. We then employed ASIC inhibitors to determine the impact of upregulated ASICs on the acidosis-induced changes. Prior experimental evidence has demonstrated that the ASIC1 specific blocker PcTx1, ASIC3 specific blocker APETx2, and the non-specific ASIC blocker Amiloride play roles in regulating the activity of NP cells [55, 57]. Here we showed that each of these ASIC inhibitors, to a lesser or greater extent, could reverse all of the acidosis-related changes in NP-MSCs. Amiloride, PcTx1 and APETx2 all reversed the decline in NP-MSC proliferative capacity and cell cycle arrest together with blocking cells from becoming senescent, providing direct evidence that targeting ASICs may be useful strategy in countering the effects of acidosis during IVDD.

Cellular senescence has been recently proposed to drive aging and age-related diseases including cancer [58]. Triggers for senescence include the constitutive DNA damage response (DDR) [58] with the downstream activation of senescence programming governed by a number of principal mediators [59] including cyclin-dependent kinase (CDK) inhibitors p21CIP1 (CDKN1A), p16INK4a (CDKN2A) and p27KIP1 (CDKN1B) [45, 60]. Previously, Human tissues and mouse models have both shown the correlation of cellular senescence with age-related disc degeneration [61–63]. Our results showed that compared to mild degenerated NP-MSCs, severe degenerated disc-derived human stem cells had higher ratio of SA-β-gal positive cells; likewise, with the increase of the induction time of acidosis, the ratio of positive cells increased. Here we showed that multiple mediators are implicated in the senescence of NP-MSCs induced by an acid microenvironment as indicated by the upregulation of the cell cycle inhibitors p27, p21 and p16. Moreover, the induction of senescence also coincided with the upregulation of p53 and Rb1, key tumor suppressor proteins associated with senescence pathways [63–68]. Instructively, the premature senescence of NP-MSCs was alleviated by ASIC inhibitors, suggesting these receptors decisively act upstream to activate senescence pathways including p53-p21/p27 and p16-Rb1 signaling.

A secondary but nonetheless important effect of acidosis concerned the effects on the cellular microenvironment. Indeed, the ECM plays a central role in the anatomical structure of the intervertebral disc and its loss during IVDD is a critical problem. Acidosis increased the expression of MMP3 and MMP9, enzymes which break down ECM components and accordingly, there were decreased levels of both collagen II and aggrecan recorded under acidic conditions. Again, Amiloride, PcTx1 and APETx2 suppressed these changes.

**Figure 9:**
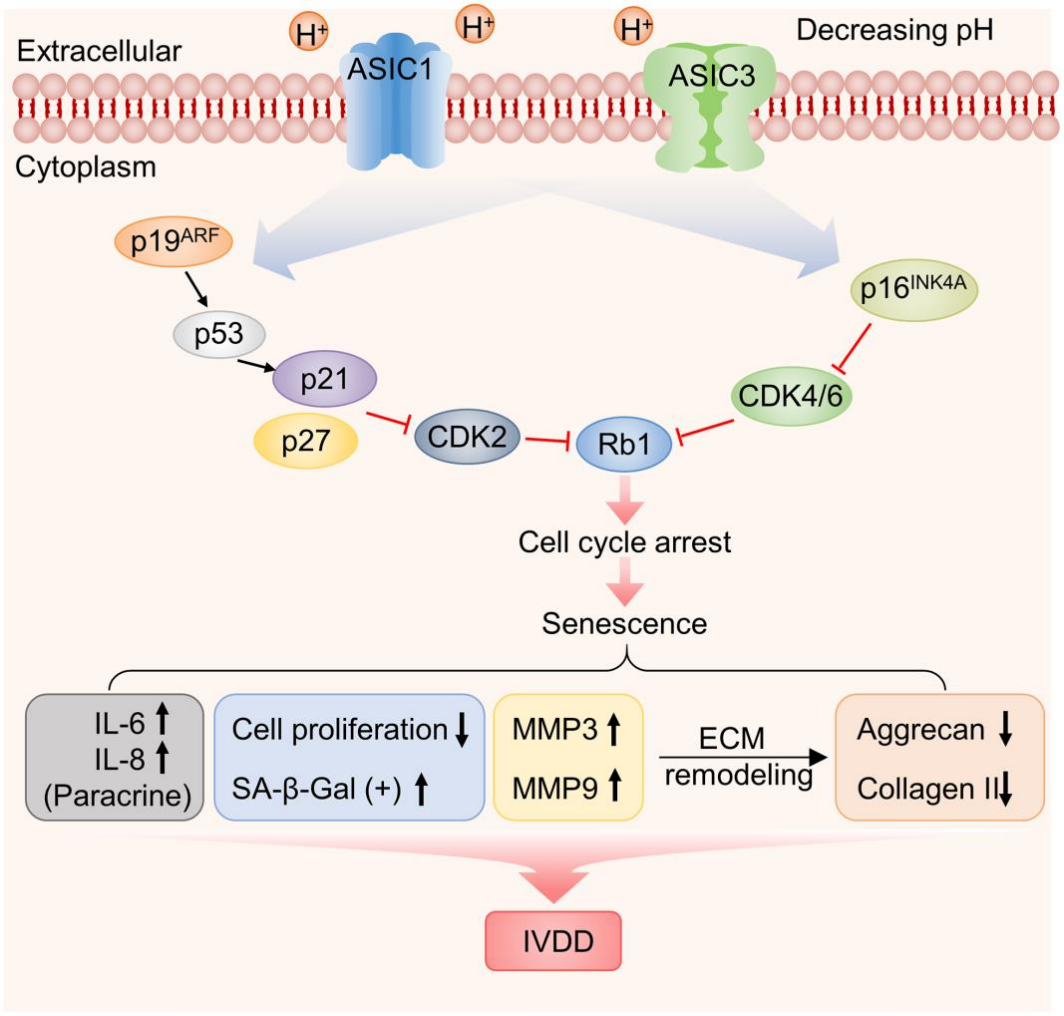
**Working model illustrating the functional contribution of ASIC1/3 expression by NP-MSCs to the acidosis-associated development of IVDD.**

In summary, our work establishes that the acidic microenvironment promotes cellular senescence in human NP-MSCs, effects mediated by ASIC1 and ASIC3 which are implicated in pleiotropic effects ranging from the induction of multiple known senescence mediators as well as effects on the integrity of the extracellular microenvironment (Figure 9). Moreover, our *in vitro* system provides a means to evaluate potential therapies such as the application and evaluation of ASIC inhibitors. Nevertheless, there are still inherent limitations, particularly that the intervertebral disc microenvironment is more complicated, and other factors are involved including hypoxia, nutritional deficiencies and low cellularity [69–71]. However, some of these aspects could readily be replicated *in vitro*. Nor have we studied the ability of NP-MSCs to transform into nucleus pulposus-like cells under acidosis conditions. From the perspective of the role of ASICs in IVDD, the precise subunit composition still needs to be defined along with a better understanding of their signaling contributions which appear from our work to be extensive.

## MATERIALS AND METHODS

### Ethics statement

Human NP tissues represent non-IVDD group were collected from 3 patients who underwent idiopathic scoliosis. The degenerative human lumbar NP specimens were collected from 9 patients with a diagnosis of lumbar disc herniation during surgical procedures to remove IVD for treatment of low back pain at the Department of Spine Surgery, the First Affiliated Hospital of Anhui Medical University. Pfirrmann grading system was used to evaluate the degree of IDD. Table 1 provides detailed information about patients age, gender and Pfirrmann classification (n=12, 7 males and 5 females; ages range from 13 to 63 years; Pfirrmann grades I, II, III and IV, 3 patients each, respectively). All human tissues were obtained with specific written informed consent with a signature from patients or their guardians with this study approved by the institutional review board of Anhui Medical University (2017372) and carried out in accordance with the relevant guidelines and regulations.

**Table 1:** Patient demographic data and degree of disc degeneration.

**Case no.**	**Age (years)**	**Gender**	**Pfirrmann grade**
Case 1	16	Male	I
Case 2	15	Male	I
Case 3	13	Female	I
Case 4	19	Male	II
Case 5	50	Female	II
Case 6	49	Female	II
Case 7	41	Female	III
Case 8	42	Male	III
Case 9	35	Male	III
Case 10	26	Female	IV
Case 11	56	Male	IV
Case 12	63	Male	IV

### Isolation of human NP-MSCs

Human NP tissue was isolated from peripheral annulus fibrous tissue and washed 3 times with sterile phosphate buffered saline (PBS, Sigma), minced carefully with aseptic ophthalmic scissors into ~0.5×0.5×0.5 mm pieces. A proportion of the minced pieces were reserved for the preparation of freshly isolated tissue homogenates while the rest were digested in 2 mg/ml type II collagenase solution (Sigma-Aldrich, USA) at 37° C for 4 hours. Following 1000×g centrifugation for 5 min, pellets (liberated cells and partially digested tissues) were collected and cultured with low-glucose Dulbecco’s modified Eagle’s medium (2 mM L-glutamine, HyClone) supplemented with 10% FBS, 1% penicillin/streptomycin in a humidified incubator at 37° C with 5% CO2. Culture medium was replaced twice a week with cells trypsinized with 0.25% Trypsin-EDTA (Biosharp) for further culture upon 80-90% confluence with subculture at 1:3 as passage 1 (P1). Cells at passage 2 were used for the *in vitro* experiments.

### Immunophenotyping of NP-MSCs

NP-MSCs from patients’ IVDs were collected by trypsinization and resuspended at 1×10^6^ cells/100 μl in PBS before immunophenotyping using flow cytometry analysis: Cells were incubated with fluorophore-conjugated monoclonal antibodies against CD45-PE, CD34-PE, HLA-DR-PE, CD73-PE, CD90-FITC and CD105-APC or isotype controls (all purchased from eBioscience, USA) at 25° C for 30 min in the dark as recommended by International Society for Cellular Therapy. The cells were washed twice with cold PBS and resuspended in 500 μl of PBS containing 1% paraformaldehyde before analysis by flow cytometry (Beckman, USA) according to standard procedures. The percentage of positive staining was calculated relative to that of the isotype control.

### Preparation of acid-conditioned medium

One M of HCl was added to adjust medium to pH 6.6 as monitored using a microelectrode (Lazarlab). Medium was equilibrated overnight in a 5% CO2 incubator at 37° C and the pH was confirmed and sterilized using a 0.04 μm filter. All acid-conditioned media (ACM) were used within one week.

### Cell proliferation assay

NP-MSC proliferation was determined using the Cell Counting Kit-8 (CCK-8, Dojindo Laboratories, Kumamoto, Japan) according to the manufacturer’s protocol. NP-MSCs were seeded at 5×10^3^ cells/well in triplicate in 96-well plates. Cells of experiment groups were cultured in ACM (pH 6.6) at 37° C with 5% CO2 except for that of the negative control group which were grown in normal media. As indicated, 100 μM Amiloride (Sigma), 20 nM PcTx1 (MCE) or 100 nM APETx2 (Abcam) was added, respectively. Cell proliferation was measured at different times by gently removing the culture media and replacing with 100 μl standard medium supplemented with 10 μl CCK-8 solution. After 4 h incubation at 37° C in the dark, the optical density (OD) values were measured at 450 nm using a SpectraMax microplate reader (Molecular Devices, USA).

### Senescence-associated β-galactosidase (SA-β-gal) staining

SA-β-gal staining was performed according to the manufacturer’s instructions after culture in 6-well plates (Beyotime, China). Briefly, after washing with PBS, cells were fixed in 1 ml of SA-β-gal fixative solution for 15 min at room temperature, rinsed three times with PBS and incubated in SA-β-gal working solution (Reagents A, B, C, and X-Gal) overnight at 37° C under atmospheric conditions. The percentage of SA-β-gal-positive cells from three randomly selected fields was counted under microscopic examination with at least 1000 cells counted for each sample.

### Cell cycle analysis

DNA content analysis was performed using the Cell Cycle Analysis Kit (BD Biosciences, USA) according to the manufacturer’s instructions. Cells were collected by trypsinization, washed twice with cold PBS and fixed with pre-chilled 75% ethanol solution overnight at -20° C. The next day, cells were pelleted by centrifugation at 1500×g for 3 min, washed twice with ice cold PBS and resuspended in 50 μl propidium iodide (PI) staining solution (BD Pharmingen, USA). After incubation for 15 min in the dark on ice, the samples were interrogated by flow cytometry using the 488 nm laser (Beckman) and the percentage of cells in the G0/G1, G2/M, and S phases determined using ModFit software (Verity Software House, Inc., Topsham, ME, USA).

### Senescence-associated secretory phenotype (SASP) assays

Secreted IL-6 and IL-8 levels were measured using the human interleukin 6 (IL-6) and interleukin-8 (IL-8/CXCL8) ELISA kits (MM-0049H1 and MM-1558H1, Jiangsu Meimian Industrial Co., Ltd) according to the recommended protocols. Briefly, primary antibodies were used to coat microtiter plate wells before the addition of 1:5 diluted samples, followed by HRP-conjugates and TMB substrate solution. Reactions were terminated by sulphuric acid solution, sample ODs measured at 450 nm and the concentrations of IL-6 and IL-8 determined against a standard curve.

### Western blotting analyses

Cell lysates were prepared using RIPA lysis butter (Beyotime P0013) and protein concentrations estimated using a Bradford protein assay (Sangon Biotech, China). Thirty μg of total protein was resolved by SDS-PAGE electrophoresis and then electro-transferred onto PVDF membranes (Bio-Rad, USA). Membranes were blocked with 5% skim milk in Tris-Buffered Saline-Tween-20 (TBST) for 1.5 h before incubating with diluted primary antibodies at 4° C overnight as follows: ASIC1 (Abcam ab240896, 1:1000), ASIC2 (Proteintech 17851-1-AP, 1:1000), ASIC3 (Alomone ASC018AN0702, 1:200), ASIC4 (Proteintech 12003-1-AP, 1:1000), p16 (Santa Cruz sc-166760, 1:500), p21 (Proteintech 10355-1-AP, 1:500), p53 (Proteintech 10442-1-AP, 1:1000), pRb1 (Proteintech 10048-2-Ig, 1:500), MMP3 (Proteintech 6338-1-Ig, 1:500), MMP9 (Proteintech 10375-2-AP, 1:500), Aggrecan (Proteintech 13880-1-AP, 1:500), Collagen II (Proteintech 15943-1-AP, 1:500) and β-actin (Proteintech 66009-1-lg, 1:10000). Antibodies were detected with the corresponding species-specific secondary antibodies at room temperature for 1 h and after washing with TBST, immunoreactive bands were observed by enhanced chemiluminescence (ECL) based detection (Thermo U1291095A). Densitometric analyses were performed using Image J with loading normalization performed against the β-actin control.

### Statistical analyses

All experiments were performed at least in triplicate with data presented as the mean ± standard deviation (SD). Student’s t test or one-way analysis of variance (ANOVA) was used to compare between treatment groups as indicated using GraphPad Prism 7 (GraphPad Software, Inc, La Jolla, CA, USA). P values < 0.05 were considered statistically significant.

## Supplementary Material

Supplementary Figures
