# Supplementary material for: ASIC1 and ASIC3 mediate cellular senescence of human nucleus pulposus mesenchymal stem cells during intervertebral disc degeneration
Source: Aging (Albany NY). 2021 Apr 6;13(7):10703–23. doi: 10.18632/aging.202850 (PMC8064223; doi:10.18632/aging.202850)
Supplement: Supplementary Figures [file aging-13-202850-s001.pdf]

SUPPLEMENTARY FIGURES

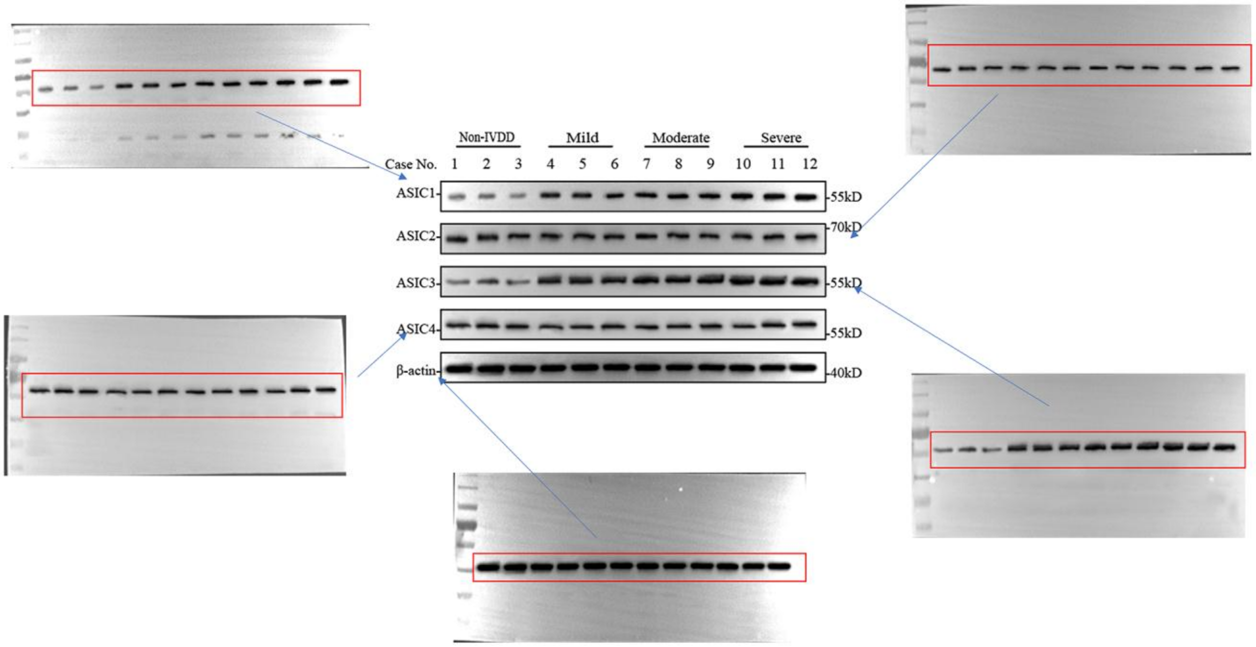

Supplementary Figure 1. Original data for Figure 2.

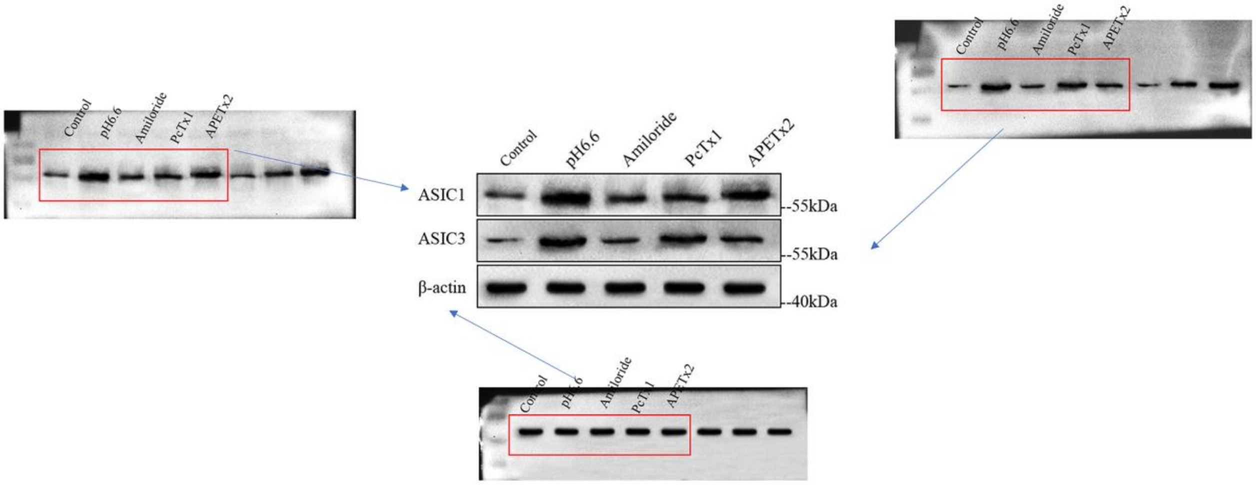

Supplementary Figure 2. Original data for Figure 3A (1).

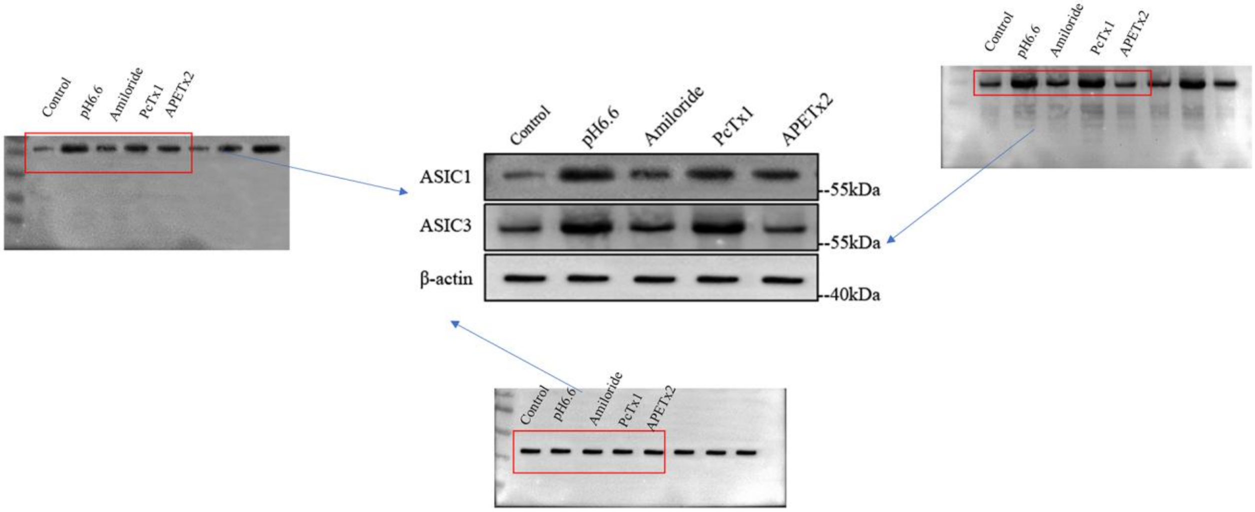

Supplementary Figure 3. Original data for Figure 3A (2).

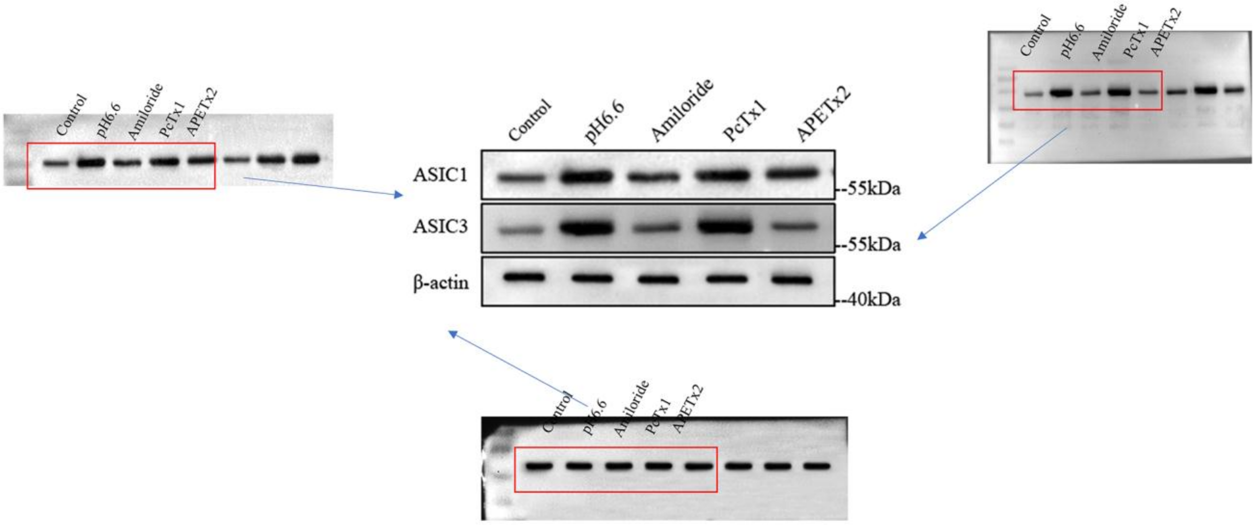

Supplementary Figure 4. Original data for Figure 3A (3).

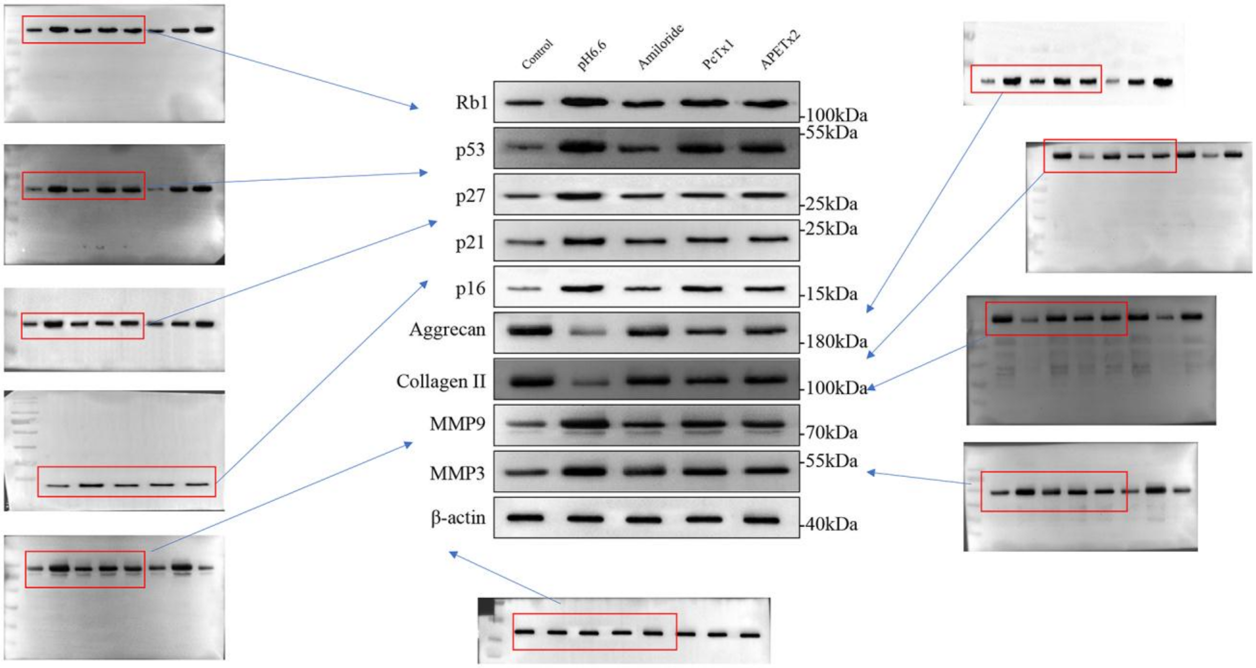

Supplementary Figure 5. Original data for Figure 8 (1).

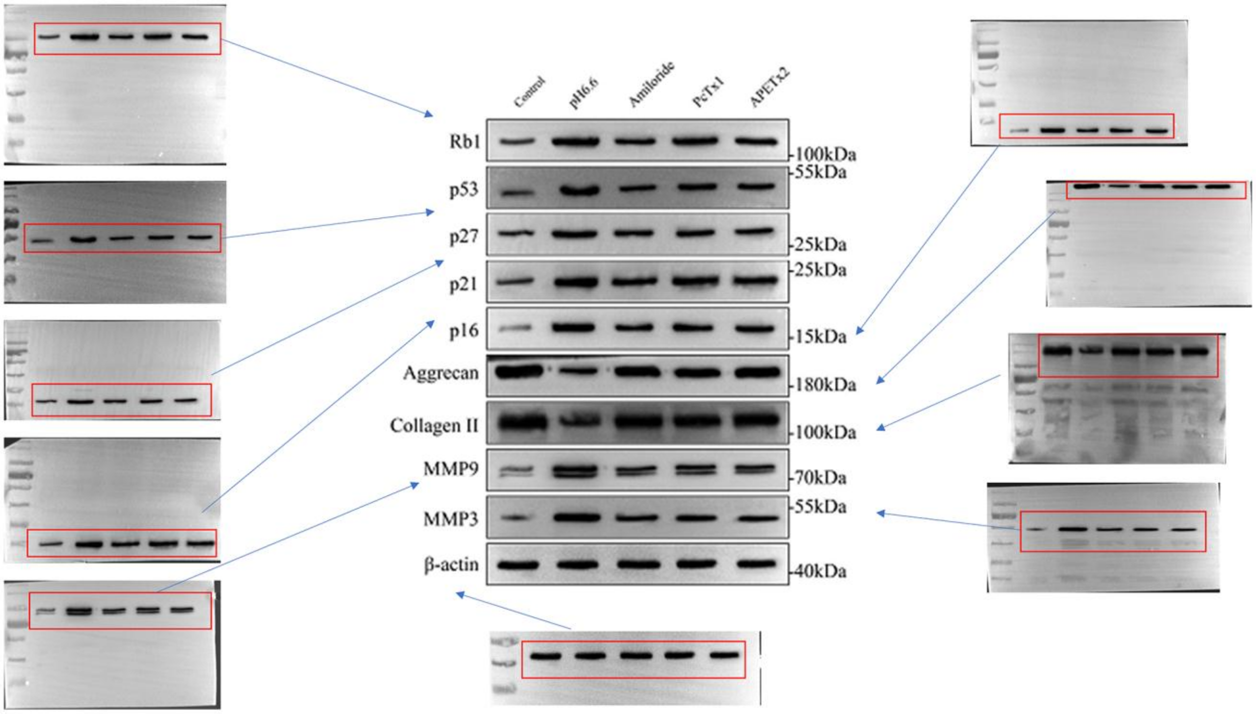

Supplementary Figure 6. Original data for Figure 8 (2).

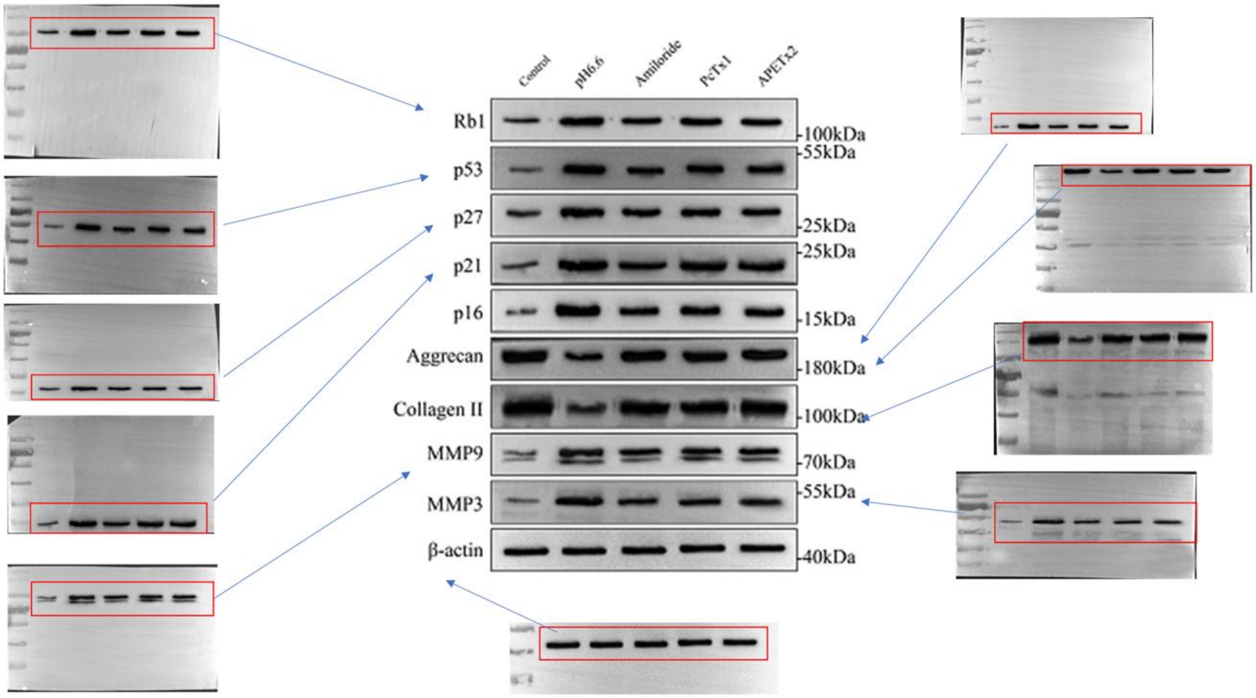

Supplementary Figure 7. Original data for Figure 8 (3).
